# Long term survival and local control outcomes from single dose targeted intraoperative radiotherapy during lumpectomy (TARGIT-IORT) for early breast cancer: TARGIT-A randomised clinical trial

**DOI:** 10.1136/bmj.m2836

**Published:** 2020-08-19

**Authors:** Jayant S Vaidya, Max Bulsara, Michael Baum, Frederik Wenz, Samuele Massarut, Steffi Pigorsch, Michael Alvarado, Michael Douek, Christobel Saunders, Henrik L Flyger, Wolfgang Eiermann, Chris Brew-Graves, Norman R Williams, Ingrid Potyka, Nicholas Roberts, Marcelle Bernstein, Douglas Brown, Elena Sperk, Siobhan Laws, Marc Sütterlin, Tammy Corica, Steinar Lundgren, Dennis Holmes, Lorenzo Vinante, Fernando Bozza, Montserrat Pazos, Magali Le Blanc-Onfroy, Günther Gruber, Wojciech Polkowski, Konstantin J Dedes, Marcus Niewald, Jens Blohmer, David McCready, Richard Hoefer, Pond Kelemen, Gloria Petralia, Mary Falzon, David J Joseph, Jeffrey S Tobias

**Affiliations:** 1 Division of Surgery and Interventional Science, University College London, 43-45 Foley Street, London W1W 7JN, UK; 2 Department of Biostatistics, University of Notre Dame, Fremantle, WA, Australia; 3 Department of Radiation Oncology, University Medical Centre Mannheim, Medical Faculty Mannheim, Heidelberg University, Heidelberg, Germany; 4 Department of Surgery, Centro di Riferimento Oncologico di Aviano (CRO) IRCCS, Aviano, Italy; 5 Department of Gynaecology and Obstetrics, Red Cross Hospital, Technical University of Munich, Munich, Germany; 6 Department of Surgery, University of California, San Francisco, CA, USA; 7 Nuffield Department of Surgical Sciences, University of Oxford, Oxford, UK; 8 School of Surgery, University of Western Australia, Crawley, WA, Australia; 9 Department of Breast Surgery, University of Copenhagen, Copenhagen, Denmark; 10 London, UK; 11 Department of Surgery, Ninewells Hospital, Dundee, UK; 12 Department of Surgery, Royal Hampshire County Hospital, Winchester, UK; 13 Department of Gynaecology and Obstetrics, University Medical Centre Mannheim, Medical Faculty Mannheim, Heidelberg University, Heidelberg, Germany; 14 Department of Radiation Oncology, Sir Charles Gairdner Hospital, Perth, WA, Australia; 15 Department of Oncology, St Olav’s University Hospital, Trondheim, Norway; 16 Department of Clinical and Molecular Medicine, Norwegian University of Science and Technology, Trondheim, Norway; 17 University of Southern California, John Wayne Cancer Institute & Helen Rey Breast Cancer Foundation, Los Angeles, CA, USA; 18 Department of Radiation Oncology, Centro di Riferimento Oncologico di Aviano (CRO) IRCCS, Aviano, Italy; 19 Instituto Oncologico Veneto, Padoa, Italy; 20 Department of Radiation Oncology, University Hospital, The Ludwig Maximilian University of Munich, Munich, Germany; 21 Radiotherapy-Oncology, Western Cancer Institute, Nantes, France; 22 Breast Centre Seefeld, Zurich, Switzerland; 23 Department of Surgical Oncology, Medical University of Lublin, Lublin, Poland; 24 Breast Centre, University Hospital Zurich, Zurich, Switzerland; 25 Saarland University Medical Center, Homberg, Germany; 26 Sankt Gertrauden Hospital, Charité, Medical University of Berlin, Berlin, Germany; 27 Princess Margaret Cancer Centre, Toronto, Ontario, Canada; 28 Sentara Surgery Specialists, Hampton, VA, USA; 29 Ashikari Breast Center, New York Medical College, New York, NY, USA; 30 Department of Surgery, University College London Hospitals, London, UK; 31 Department of Pathology, University College London Hospitals, London, UK; 31 Department of Clinical Oncology, University College London Hospitals, London, UK

## Abstract

**Objective:**

To determine whether risk adapted intraoperative radiotherapy, delivered as a single dose during lumpectomy, can effectively replace postoperative whole breast external beam radiotherapy for early breast cancer.

**Design:**

Prospective, open label, randomised controlled clinical trial.

**Setting:**

32 centres in 10 countries in the United Kingdom, Europe, Australia, the United States, and Canada.

**Participants:**

2298 women aged 45 years and older with invasive ductal carcinoma up to 3.5 cm in size, cN0-N1, eligible for breast conservation and randomised before lumpectomy (1:1 ratio, blocks stratified by centre) to either risk adapted targeted intraoperative radiotherapy (TARGIT-IORT) or external beam radiotherapy (EBRT).

**Interventions:**

Random allocation was to the EBRT arm, which consisted of a standard daily fractionated course (three to six weeks) of whole breast radiotherapy, or the TARGIT-IORT arm. TARGIT-IORT was given immediately after lumpectomy under the same anaesthetic and was the only radiotherapy for most patients (around 80%). TARGIT-IORT was supplemented by EBRT when postoperative histopathology found unsuspected higher risk factors (around 20% of patients).

**Main outcome measures:**

Non-inferiority with a margin of 2.5% for the absolute difference between the five year local recurrence rates of the two arms, and long term survival outcomes.

**Results:**

Between 24 March 2000 and 25 June 2012, 1140 patients were randomised to TARGIT-IORT and 1158 to EBRT. TARGIT-IORT was non-inferior to EBRT: the local recurrence risk at five year complete follow-up was 2.11% for TARGIT-IORT compared with 0.95% for EBRT (difference 1.16%, 90% confidence interval 0.32 to 1.99). In the first five years, 13 additional local recurrences were reported (24/1140 *v* 11/1158) but 14 fewer deaths (42/1140 *v* 56/1158) for TARGIT-IORT compared with EBRT. With long term follow-up (median 8.6 years, maximum 18.90 years, interquartile range 7.0-10.6) no statistically significant difference was found for local recurrence-free survival (hazard ratio 1.13, 95% confidence interval 0.91 to 1.41, P=0.28), mastectomy-free survival (0.96, 0.78 to 1.19, P=0.82), distant disease-free survival (0.88, 0.69 to 1.12, P=0.30), overall survival (0.82, 0.63 to 1.05, P=0.13), and breast cancer mortality (1.12, 0.63 to 1.28, P=0.54). Mortality from other causes was significantly lower (0.59, 0.40 to 0.86, P=0.005).

**Conclusion:**

For patients with early breast cancer who met our trial selection criteria, risk adapted immediate single dose TARGIT-IORT during lumpectomy was an effective alternative to EBRT, with comparable long term efficacy for cancer control and lower non-breast cancer mortality. TARGIT-IORT should be discussed with eligible patients when breast conserving surgery is planned.

**Trial registration:**

ISRCTN34086741, NCT00983684.

## Introduction

In 2018, two million patients were diagnosed as having breast cancer worldwide and 626 000 patients died from the disease.^1^ Treatment with breast conserving surgery and adjuvant radiotherapy rather than total mastectomy is suitable for most patients. Most local recurrences occur close to the primary tumour site despite the frequent presence of microscopic cancer foci in other quadrants.^2^ ^3^ Based on the hypothesis that adjuvant radiotherapy for women with early breast cancer could be limited to the tumour bed and given immediately during breast conserving surgery (lumpectomy), we developed the concept of targeted intraoperative radiotherapy (TARGIT-IORT).^4^ ^5^ ^6^ ^7^ When the TARGIT-A trial protocol was published in 1999,^8^ restricting radiotherapy to only the area around the tumour had been explored in small patient series^9^ and one randomised trial,^10^ which had reported inferior results. At that time whole breast radiotherapy was the standard of care, and it remains so today, despite the publication of our initial results^11^ ^12^ ^13^ and several other approaches.^14^ ^15^ ^16^ ^17^ ^18^ ^19^ ^20^ ^21^

TARGIT-IORT provides a well positioned and rapid form of tumour bed irradiation focused on the target tissues alone, while sparing normal tissues and organs such as heart, lung, skin, and chest wall structures.^22^ We designed the TARGIT-A randomised trial to compare risk adapted TARGIT-IORT with conventional whole breast external beam radiotherapy (EBRT) over several weeks.^4^ ^11^ ^13^ The study received ethics approval from the Joint University College London and University College London Hospital committees of ethics of human research. Recruitment began in March 2000 and was completed in June 2012.

In 2004, four years after recruitment began for the main TARGIT-A trial and at the request of centres with potentially high numbers of patients, we sought additional ethics approval and opened a parallel study. This study was previously referred to as the post-pathology stratum and recruited 1153 patients by using a separate randomisation table. These patients were randomised after their initial surgery to have either conventional fractionated whole breast radiotherapy (n=572) or a further operation to deliver delayed radiotherapy to the wound by reopening the original incision (n=581). The trial was initiated mainly because of easier scheduling of delayed TARGIT-IORT in operating theatres. This delayed radiotherapy was not intraoperative radiotherapy given during the cancer operation; treatment was performed a median of 37 days after the first excision. The 2013 analysis found that this delayed second procedure crossed the 2.5% margin of non-inferiority. Therefore, we recommended that immediate TARGIT-IORT should be the preferred treatment over delayed TARGIT-IORT,^13^ and delayed treatment was no longer used. As specified in the statistical analysis plan, which was signed off before unblinding for this analysis, we have addressed the long term outcomes for this parallel trial in a separate paper. 

This paper reports the findings of the TARGIT-A trial, in which 2298 patients were randomised after their needle biopsy and before any surgical excision of the cancer to receive either risk adapted TARGIT-IORT delivered during the initial excision of cancer or postoperative whole breast external beam radiotherapy (EBRT). We investigated whether immediate TARGIT-IORT was non-inferior to EBRT at five year complete follow-up in terms of local recurrence, and also compared their long term survival outcomes.

## Methods

TARGIT-A was a pragmatic, prospective, international, multicentre, open label, randomised, phase III trial that compared risk adapted TARGIT-IORT with the conventional treatment of whole breast EBRT. The trial protocol (https://njl-admin.nihr.ac.uk/document/download/2006598), including details of sample size calculations and the random allocation process, has been previously described.^11^ ^13^ In brief, women with early breast cancer were eligible if they were aged 45 years or older, diagnosed by needle biopsy, and suitable for wide local excision of invasive ductal carcinoma that was unifocal on conventional examination and imaging (cT1 and small cT2 ≤3.5 cm, cN0-N1, M0, as confirmed by cytology or histology). Breast magnetic resonance imaging was not required and only 5.6% of patients in the trial had a scan.

Eligible patients were randomly assigned before their surgery (in a 1:1 ratio) to receive either a risk adapted approach that used single dose TARGIT-IORT or EBRT according to standard schedules over several weeks, with randomisation blocks stratified by centre. The randomisation schedules were generated centrally by computer (securely kept in trial centres in Perth for Australian centres and London, United Kingdom, for all other centres). Requests for randomisation were through telephone or fax to the trial office (Perth or London), where a trained member of staff checked patient eligibility. Treatment was allocated from a preprinted randomisation schedule available to authorised staff only. Written confirmation of randomisation was sent by fax to the site.

All patients gave written informed consent and needed to be available for regular follow-up for at least 10 years. Follow-up clinical examination was at least six monthly for the first five years and annually thereafter, including a mammogram once a year.

The experimental arm was risk adapted radiotherapy. If the final pathology report showed prespecified unpredicted features, EBRT was recommended in addition to TARGIT-IORT, with TARGIT-IORT (already received during surgery) serving as the tumour bed boost. In the core protocol, EBRT was recommended to supplement TARGIT-IORT within the experimental arm if the tumour-free margin was less than 1 mm, if there was an extensive in situ component (>25%), or if unexpected invasive lobular carcinoma was found in the postoperative final microscopic histopathological examination of the primary tumour excision. Additionally, individual centres prespecified any other final postoperative histopathology criteria (such as grade 3 tumour, node positivity, lymphovascular invasion) that would prompt supplemental EBRT to be recommended. These criteria were recorded in the centre’s treatment policy document before their trial recruitment started.

The trial was a comparison of two policies: whole breast radiotherapy without selection versus individualised risk adapted radiotherapy; a proportion of patients who received TARGIT-IORT were also given supplemental EBRT by using prespecified criteria. These patients were not crossovers, but were offered individualised risk adapted radiotherapy according to the experimental treatment policy, which was designed to reflect the real world scenario.

The TARGIT-IORT technique using the Intrabeam device (Carl Zeiss Meditec, Oberkochen, Germany)^5^ ^6^ ^7^ enables a patient to potentially receive all the required radiation in a single treatment under the same anaesthetic as the primary surgery (efig 1).^5^ ^6^ ^7^ ^23^ ^24^ ^25^ ^26^ Radiation is delivered from a point source of 50 kV energy x rays at the centre of a spherical applicator over 20-50 minutes. The appropriately sized (1.5-5 cm diameter) applicator is surgically positioned in the tumour bed so that breast tissues at risk of local recurrence receive the prescribed dose while skin and other organs are protected. The surface of the tumour bed typically receives 20 Gy that attenuates to 5-7 Gy at 1 cm depth. Further details and a video are available online (www.targit.org.uk; https://goo.gl/iuF9ZR). The patients in the conventional arm underwent standard EBRT which always included fractionated whole breast radiotherapy for three to six weeks, with or without an EBRT tumour bed boost, as determined by local criteria prespecified by the collaborating centre.

We designed the trial as a non-inferiority trial. Non-inferiority trials in cancer are performed to test new treatments that have obvious non-oncological advantages, such as better access, convenience, or quality of life for the patient, or reduced costs for the healthcare system. The non-inferiority statistical test for such a comparison is not meant to check for superiority, but to assess if the difference is within an acceptable margin and the experimental treatment is not meaningfully worse than the control. Therefore, whether the difference seen between the two randomised arms is statistically significant is not relevant here. As long as the absolute difference is not clinically significant, the new treatment would be deemed non-inferior.^27^ Any chosen non-inferiority margin must be one that clinicians and patients agree is an acceptable difference for the sake of the other benefits. These benefits might include lower toxicity, better cosmetic outcome, better quality of life, and overall patient preference. Therefore, in the original protocol, non-inferiority was specified as being achieved if the difference in the binomial proportions of local recurrence rate at five years did not cross a stringent margin of 2.5% in absolute terms; that is, local recurrence risk with TARGIT-IORT minus local recurrence risk with EBRT should not be more than 0.025 (2.5%). In the 2013 analysis, an even more rigorous criterion was used, specifying that the upper 90% confidence interval of the absolute difference must not exceed 0.025 (2.5%).

The 2.5% non-inferiority margin in the TARGIT-A trial is a relevant, relatively stringent margin. Patient preference studies in the United States, Australia, and Europe suggest that 2.5% is an acceptable margin.^28^ ^29^ ^30^ Importantly, it is widely regarded as a safe margin because it is well established that a local recurrence difference of less than 10% at five years does not worsen breast cancer survival^31^; that is, when the risk in arm A minus the risk in arm B is less than 0.1 (10%). A large increase in local recurrences (>10% at five years) is required to lead to increased mortality because they can be effectively treated. For example, a 20% increase in local recurrence (a risk increase by 0.2) would cause a 5% increase in deaths (a mortality risk increase by 0.05)^31^; this was the basis for the ethics approval of this trial. In the ELIOT trial, which also investigated intraoperative radiotherapy, the non-inferiority margin was set at 7%.^15^ In recently reported trials of systemic therapy, the margin of a 3% difference in disease-free survival was considered acceptable.^32^

Analysis of conventional longer term outcomes in breast cancer trials needs to include deaths as events for two reasons. Firstly, deaths are one of the most important clinical outcomes. Secondly, longer follow-up in an older population with early breast cancer means that death becomes much more common than local recurrence. Importantly, if toxicity of treatment leads to a difference in mortality then it needs to be reflected in the results. The statistical analysis plan for this long term analysis was signed off by the chair of the independent steering committee and an independent senior statistician before the unblinded data were sent to the trial statistician for the current analysis. The plan specified the primary outcome was local recurrence-free survival. This outcome is consistent with the DATECAN^33^ and STEEP^34^ guidelines for clinical events to be included in the definitions of time-to-event end points in randomised clinical trials assessing treatments for breast cancer. Local recurrence-free survival is clinically meaningful because it measures the chance of a patient being alive without local recurrence. Therefore, this outcome includes local recurrence or death as events; that is, patients who had died were not censored. Clinicians and patients need to know the chance of being alive without a local recurrence, which is given by local recurrence-free survival. The other important outcomes were invasive local recurrence-free survival, mastectomy-free survival, distant disease-free survival, overall survival, breast cancer mortality, and non-breast cancer mortality.

We performed statistical analysis by using established methods.^27^ ^35^ ^36^ Hazard ratios were calculated by using the Cox proportional hazard model with TARGIT-IORT as the numerator. We carried out censoring appropriately for each outcome; for example, for survival outcomes, patients were censored at the time of last follow-up, or the date of withdrawal. Kaplan-Meier graphs for these long term outcomes were presented according to Pocock and colleagues,^37^ who recommended that the x axis should be extended until 10-20% of patients are at risk of an event. This approach also ensures that any long term trends (positive or negative) are not missed. We used the log rank test to compare differences between survival functions and to obtain P values. All analyses were by intention to treat according to the randomisation arm.

Each centre specified the cause of death. If the cause of death was specified as a non-breast cancer event and no distant disease was recorded, it was defined as a non-breast cancer death. If the death was recorded by the centre to be related to breast cancer, or as per convention, if breast cancer was present at the time of death, or if the cause of death was recorded as unknown or uncertain, it was presumed to be a breast cancer death.

The reference date for completeness was 2 May 2018, eight years after the first data lock. We considered patients to have complete follow-up if they were seen for the specified duration of follow-up, if they were seen within one year of the reference date, if they had died, or if they had withdrawn from the trial. Because the last patient was randomised in 2012, the statistical analysis plan specified that the five year follow-up would be considered complete if 95% of patients had complete follow-up. The plan also specified that a 10 year follow-up would be considered complete if the patient had at least 10 years of follow-up, had been seen within one year of the reference date, or had died or withdrawn from the study; the 10 year follow-up would be considered complete if this was achieved by 90% of patients. The interim analysis confirmed the safety of TARGIT-IORT, but the follow-up was relatively short. Therefore, the independent data monitoring committee recommended that we continue recruitment while accruing the required follow-up. There was no specific trial funding for individual centres and so return of follow-up relied on individual investigators and the efforts of their teams, enthused by the trial centre team. The trial statistician and the chief investigator produced reports of completeness of follow-up by using blinded databases on a regular basis.

Once the thresholds set in the statistical analysis plan were reached, the database was unblinded for analysis. The reference date for analysis was 3 July 2019, so that all events up until 2 July 2019 were included for analysis. We used Stata version 16.0 for data compilation, validation, and analysis. The trial steering and data monitoring committees each included a patient advocate as a member. Since the last analysis, the trial oversight has been provided by an independent steering committee, appointed by the Health Technology Assessment (HTA) programme of the National Institute of Health Research, Department of Health and Social Care, UK, which also includes a patient as a member.

### Patient and public involvement

Patients have been involved as members of the steering committee from the start. Patients were not involved in the initial design of the study in 1999-2000, but they were involved from the time the trial started. However, it was the serious concern about patients’ welfare that inspired the study design. The pragmatic nature of this trial was designed to suit the patient’s perspective. The non-inferiority margin has been validated with patient preference studies,^28^ ^29^ ^30^ which included asking patients about their priorities. Patients were involved in recruitment to and conduct of the study as members of the steering committee and on several occasions as commentators in the national press, TV, and radio. Patients assessed the burden of intervention and the time required to participate. Unlike most other studies, participating in the trial was the main pathway through which patients could access TARGIT-IORT and reduce the burden of treatment (that is, they were likely to avoid external beam radiotherapy) in the 50% of the group randomised to receive the TARGIT-IORT arm rather than the EBRT arm. A patient has been involved during the development of the statistical analysis plan, interpretation of the results and writing of the manuscript, and is an author of the paper.

## Results

Between 24 March 2000 and 25 June 2012, 2298 patients were recruited to the study: 1140 patients were randomised to receive risk adapted immediate TARGIT-IORT during lumpectomy and 1158 patients were randomised to receive EBRT. Table 1 presents patient and tumour characteristics, which were well matched between the randomised arms.

**Table 1:** Patient and tumour characteristics in the TARGIT-IORT and EBRT arms

Characteristics	TARGIT-IORT (n=1140)	EBRT (n=1158)
**Age (years)**		
≤50	117 (10.3)	99 (8.6)
51-60	362 (31.8)	375 (32.4)
61-70	481 (42.2)	524 (45.3)
>70	180 (15.8)	160 (13.8)
**Body mass index **		
Normal (<25)	408 (41.0)	420 (42.2)
Overweight (25-29.9)	375 (37.7)	329 (33.1)
Obese (≥30)	212 (21.3)	246 (23.7)
Unknown	145 (12.7)	163 (14.1)
**Specimen weight (g)**		
Median (interquartile range)	40 (25-65)	40 (24-70)
**Pathological tumour size (mm; P=0.70)**		
≤10	369 (33.1)	370 (33.1)
11-20	571 (51.2)	557 (49.9)
>20	176 (15.8)	190 (17.0)
Unknown	24 (2.1)	41 (3.5)
**Grade**		
1	275 (24.5)	286 (25.6)
2	621 (55.4)	615 (55.0)
3	226 (20.1)	217 (19.4)
Unknown	18 (1.6)	40 (3.5)
**Margin**		
Free	1007 (89.4)	993 (88.2)
Ductal carcinoma in situ only	54 (4.8)	60 (5.3)
Invasive	65 (5.8)	73 (6.5)
Unknown	14 (1.2)	32 (2.8)
**Re-excision**	76 (6.7)	97 (8.4)
**Lymphovascular invasion**		
Absent	931 (83.4)	946 (84.6)
Present	185 (16.6)	172 (15.4)
Unknown	24 (2.1)	40 (3.5)
**Lymph nodes involved**		
0	872 (77.4)	893 (79.2)
1-3	213 (18.9)	205 (18.2)
>3	41 (3.6)	29 (2.6)
Unknown	14 (1.2)	31 (2.7)
**Estrogen receptor status**		
Positive	1005 (89.8)	1030 (91.7)
Negative	114 (10.2)	93 (8.3)
Unknown	21 (1.8)	35 (3.0)
**Progesterone receptor status**		
Positive	895 (80.3)	921 (82.7)
Negative	220 (19.7)	193 (17.3)
Unknown	25 (2.2)	44 (3.8)
**Human epidermal growth factor receptor 2 status**		
Positive	156 (14.5)	164 (15.1)
Negative	920 (85.5)	925 (84.9)
Unknown	64 (5.6)	69 (6.0)
**Method of presentation**		
Screen detected	739 (67.0)	755 (68.0)
Symptomatic	364 (33.0)	355 (32.0)
Unknown	37 (3.3)	48 (4.2)
**Endocrine therapy**		
Received	897 (81.5)	894 (81.1)
Did not receive	204 (18.5)	209 (18.9)
Unknown	39 (3.4)	55 (4.8)
**Chemotherapy**		
Received	239 (21.7)	218 (19.7)
Did not receive	863 (78.3)	887 (80.3)
Unknown	38 (3.3)	53 (4.6)

EBRT=external beam radiotherapy; TARGIT-IORT=targeted intraoperative radiotherapy.

Data are numbers (percentages). For percentage calculation, the denominator for unknown percentages is the total number randomised (1140 and 1158) and the denominator for each category is the total number of known patients. No imbalance was found for any of these characteristics between the two randomised arms.

**Fig 1:**
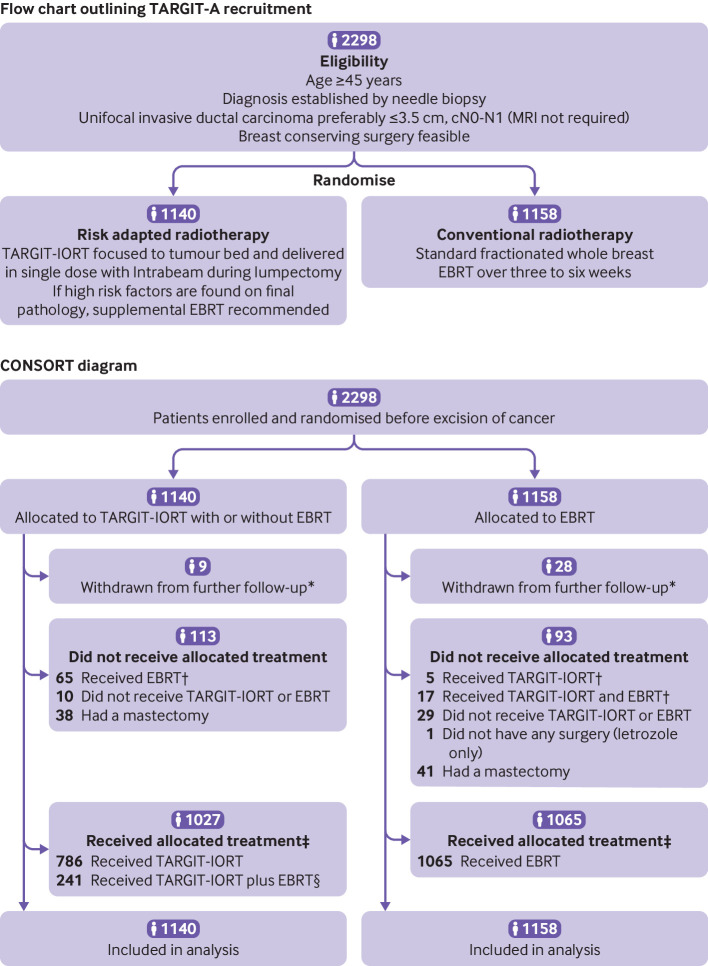
Flowchart outlining TARGIT-A recruitment and CONSORT (consolidated standards of reporting trials) diagram. *Difference in number withdrawn was statistically significant (P=0.002). †Crossovers: 65/1140 (5.7%) allocated TARGIT-IORT received EBRT, and 22/1158 (1.9%) allocated EBRT received TARGIT-IORT. ‡1027/1140 (91%) allocated TARGIT-IORT and 1065/1158 (92%) allocated EBRT received allocated treatment. §As per protocol, 241/1140 (21.1%) patients allocated TARGIT-IORT received EBRT after TARGIT-IORT. EBRT=external beam radiotherapy; TARGIT-IORT=targeted intraoperative radiotherapy

**Fig 2:**
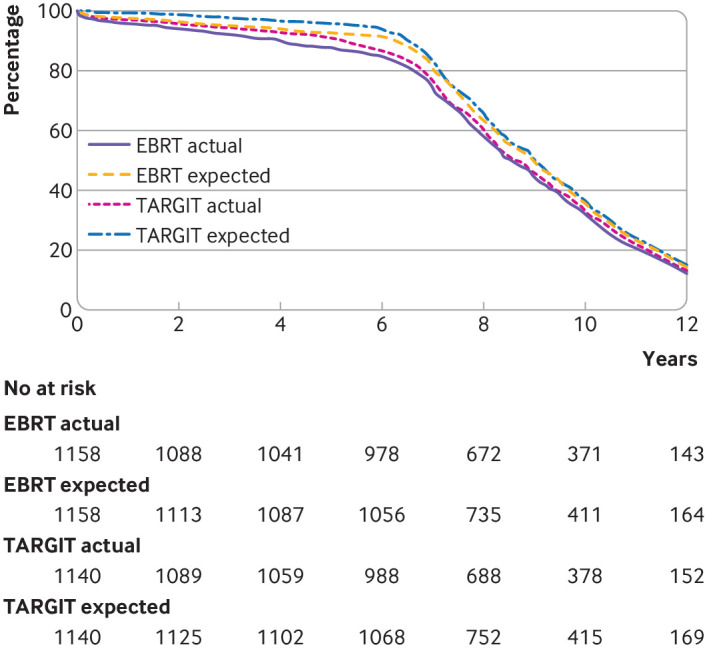
Completeness of follow-up. Curves for actual follow-up and how close they are to curves for expected follow-up. Expected is presumed equal to actual if patients have withdrawn or died. No significant difference in follow-up duration between TARGIT-IORT and EBRT (log rank P=0.22). EBRT=external beam radiotherapy; TARGIT-IORT=targeted intraoperative radiotherapy

Complete follow-up to the prespecified level of 95% at five years was achieved by mid-2019. Figure 1 presents the flow and CONSORT (consolidated standards of reporting trials) diagrams. Figure 2 shows the completeness of follow-up and illustrates that the observed follow-up is close to the expected follow-up in each arm of the trial. The follow-up duration of the two arms did not differ (log rank P=0.22).

For the protocol specified primary outcome of non-inferiority at five years, we found that immediate TARGIT-IORT was non-inferior to EBRT for local control (table 2): at five year complete follow-up, the number of local recurrences was 24 (including six ductal carcinoma in situ) of 1140 (2.11%) for TARGIT-IORT versus 11 (including one ductal carcinoma in situ) of 1158 (0.95%) for EBRT. The difference in local recurrence rate was 0.0116 (1.16%) and the 90% confidence interval was 0.0032 to 0.0199 (0.32% to 1.99%), establishing non-inferiority. Testing for non-inferiority by using five year Kaplan-Meier estimates also confirmed that immediate TARGIT-IORT is non-inferior to EBRT (difference 1.21%, 90% confidence interval 0.47% to 1.95%). We also confirmed non-inferiority when 95% confidence intervals were used, and when per protocol analysis was performed with 90% and 95% confidence intervals. The number of deaths was 42 of 1140 for TARGIT-IORT versus 56 of 1158 for EBRT.

**Table 2:** Analysis of non-inferiority by using binomial proportions and Kaplan-Meier estimates

Analysis	TARGIT-IORT	EBRT
**Intention-to-treat analysis (n=1140; n=1158)**		
Binomial proportions of five year local recurrence	2.11	0.95
Difference (90% CI; 95% CI)	1.16 (0.32 to 1.99; 0.15 to 2.16)	
Kaplan-Meier estimates of local recurrence at five year complete follow-up (SE)	2.23 (0.45)	1.02 (0.31)
Difference (90% CI; 95% CI)	1.21 (0.47 to 1.95; 0.33 to 2.09)	
**Per protocol analysis (n=1027; n=1065)**		
Binomial proportions of five year local recurrence	2.24	0.94
Difference (90% CI; 95% CI)	1.30 (0.40 to 2.20; 0.23 to 2.38)	
Kaplan-Meier estimates of local recurrence at five year complete follow-up (SE)	2.36 (0.49)	0.99% (0.31)
Difference (90% CI; 95% CI)	1.37 (0.56 to 2.18; 0.41 to 2.33)	

EBRT=external beam radiotherapy; SE=standard error; TARGIT-IORT=targeted intraoperative radiotherapy.

The protocol specified that the non-inferiority test should be performed with a prespecified margin of 2.5% at five years. The statistical analysis plan stated that analysis of non-inferiority should be performed by calculating difference in binomial proportion of local recurrence rates at five years, and that non-inferiority would be considered as established if upper 90% confidence interval of difference did not cross 0.025 (2.5%). Local recurrence risk and difference in this risk are depicted as absolute percentage (eg, difference in risk of 0.0116 is depicted as 1.16%). For completeness, test for non-inferiority was performed by using five year Kaplan-Meier estimates of local recurrence and per protocol analysis. Results show that TARGIT-IORT remains non-inferior to EBRT.

**Fig 3:**
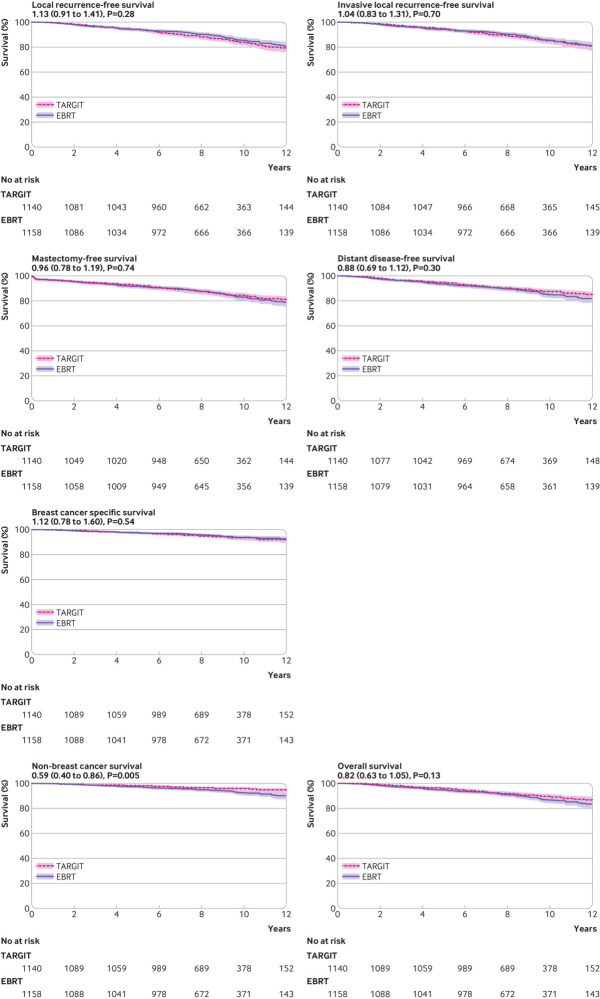
Kaplan-Meier estimates and curves for the following outcomes for TARGIT-IORT versus EBRT in the TARGIT-A trial: local recurrence-free survival, invasive local recurrence-free survival, mastectomy-free survival, distant disease-free survival, breast cancer specific survival, non-breast cancer survival, and overall survival. Figures under titles are hazard ratios (95% confidence intervals) and log rank test P values. EBRT=external beam radiotherapy; TARGIT-IORT=targeted intraoperative radiotherapy

**Fig 4:**
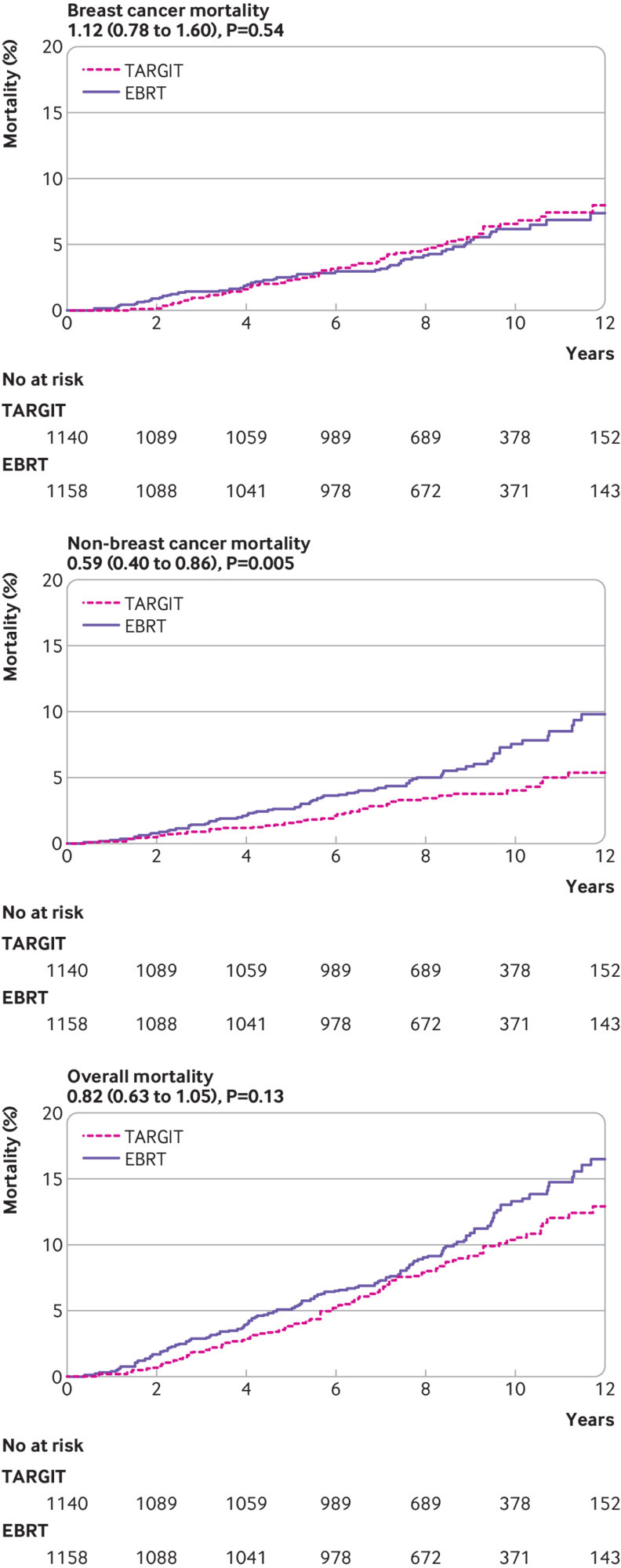
Kaplan-Meier curves showing differences in breast cancer mortality, non-breast cancer mortality, and overall mortality in TARGIT-A trial for TARGIT-IORT *v* EBRT. Figures under titles are hazard ratios (95% confidence intervals) and log rank test P values. EBRT=external beam radiotherapy; TARGIT-IORT=targeted intraoperative radiotherapy

With long term follow-up (median 8.6 years, maximum 18.9 years, interquartile range 7.0-10.6), no statistically significant difference was found between immediate TARGIT-IORT and EBRT for the following outcomes: local recurrence-free survival (167 *v* 147 events, hazard ratio 1.13, 95% confidence interval 0.91 to 1.41, P=0.28), invasive local recurrence-free survival (154 *v* 146 events, 1.04, 0.83 to 1.31, P=0.70), mastectomy-free survival (170 *v* 175 events, 0.96, 0.78 to 1.19, P=0.82), distant disease-free survival (133 *v* 148 events, 0.88, 0.69 to 1.12, P=0.30), overall survival (110 *v* 131 events, 0.82, 0.63 to 1.05, P=0.13), and breast cancer mortality (65 *v* 57 events, 1.12, 0.63 to 1.28, P=0.54). Mortality from other causes was significantly lower (45 *v* 74 events, 0.59, 0.40 to 0.86, P=0.005). Analysis according to treatment received found that local recurrence-free survival was no different from EBRT for the following comparisons: TARGIT-IORT plus EBRT (n=241) versus EBRT (n=1065): hazard ratio 1.25, 95% confidence interval 0.87 to 1.80, P=0.24; and TARGIT-IORT alone (n=786) versus EBRT (n=1065): 1.22, 0.95 to 1.57, P=0.11. We used Schoenfeld residuals to confirm that the proportionality assumption was not violated (P=0.87 for local recurrence-free survival and P=0.81 for mortality). We also confirmed that there was no heterogeneity between countries (efig 2). The number of patients who died with uncontrolled local recurrence at the time of death was similar in the two arms of the trial (4/1140 for TARGIT-IORT and 5/1158 for EBRT, P=0.76). Table 3 gives the number of events and absolute event rates for local recurrence and mortality up to five years, and beyond five years. Figure 3 shows the Kaplan-Meier curves and figure 4 shows magnified Kaplan-Meier curves. Table 4 gives the causes of death.

**Table 3:** Number of events and absolute event rates (percentages) of local recurrence and death

Local recurrence and death	TARGIT-IORT (n=1140)			EBRT (n=1158)	
	≤5 years	>5-19 years		≤5 years	>5-19 years
Local recurrence was invasive with or without DCIS	15 (1.3)	17 (1.5)		9 (0.8)	10 (0.9)
Local recurrence was only DCIS	6 (0.5)	6 (0.6)		1 (0.1)	0
Local recurrence type was unknown* (assumed as invasive for analysis)	3 (0.3)	13 (1.1)		1 (0.1)	3 (0.3)
No of deaths	42 (3.7)	68 (5.9)		56 (4.8)	75 (6.5)

DCIS=ductal carcinoma in situ; EBRT=external beam radiotherapy; SE=standard error; TARGIT-IORT=targeted intraoperative radiotherapy.

There is complete follow-up at five years and maximum follow-up at 18.9 years. This table gives the number of events up to five years and beyond five years. The protocol specified that number of local recurrences (all types) at five years should be used for calculation of non-inferiority at 2.5% margin and all types of local recurrences were included.

* Local recurrence of unknown type was included as invasive local recurrence in long term invasive local recurrence-free survival analysis.

**Table 4:** Number of deaths from breast cancer and other causes

Causes of death	TARGIT-IORT	EBRT	Total
**Death from breast cancer**			
Breast cancer*	35	32	—
Breast cancer present at time of death*	6	7	—
Unknown or uncertain*	24	18	—
Total breast cancer deaths	65	57	122
Death from other causes*			
Other cancers	15	21	—
Cardiovascular causes	8	20	—
Pulmonary causes	4	9	—
Other causes/exact cause not given	18	24	—
Total non-breast cancer deaths	45	74	119
**Total**	110	131	241

EBRT=external beam radiotherapy; TARGIT-IORT=targeted intraoperative radiotherapy.

* Case record form for death completed by centre stipulated classification of deaths as one of the following: breast cancer; breast cancer present at time of death including previously reported distant disease; not breast cancer and breast cancer not present; unknown or uncertain. As per convention, only deaths classified as not breast cancer and breast cancer not present were classified as non-breast cancer deaths.

## Discussion

We based the TARGIT-IORT approach on the clinical observation that local recurrence after breast conserving surgery, with or without whole breast irradiation, occurs predominantly within the index quadrant.^2^ ^3^ This observation holds true despite the fact that more than 60% of patients for whom breast conservation is a treatment have foci of the disease outside the index quadrant.^2^ ^3^ ^38^ Using this observation that most local recurrences occur in the index quadrant as the rationale for partial breast irradiation has also been reiterated by subsequent investigators.^15^ ^17^ ^19^ The propensity of tumour recurrence in the index quadrant could be owing to a tumour promoting effect of the microenvironment of the surgical wound,^39^ ^40^ ^41^ a risk that seems to be favourably influenced by TARGIT-IORT to the fresh tumour bed.^39^ ^41^ ^42^

Early results of using single dose TARGIT-IORT during lumpectomy were promising, and the treatment was found to have advantages for the patient, such as convenience, reduced travel and personal costs, improved quality of life, and fewer side effects.^43^ ^44^ ^45^ ^46^ ^47^ However, the international community has been waiting for the long term follow-up outcomes before this approach is more widely adopted.

### Statement of principal findings

**Fig 5:**
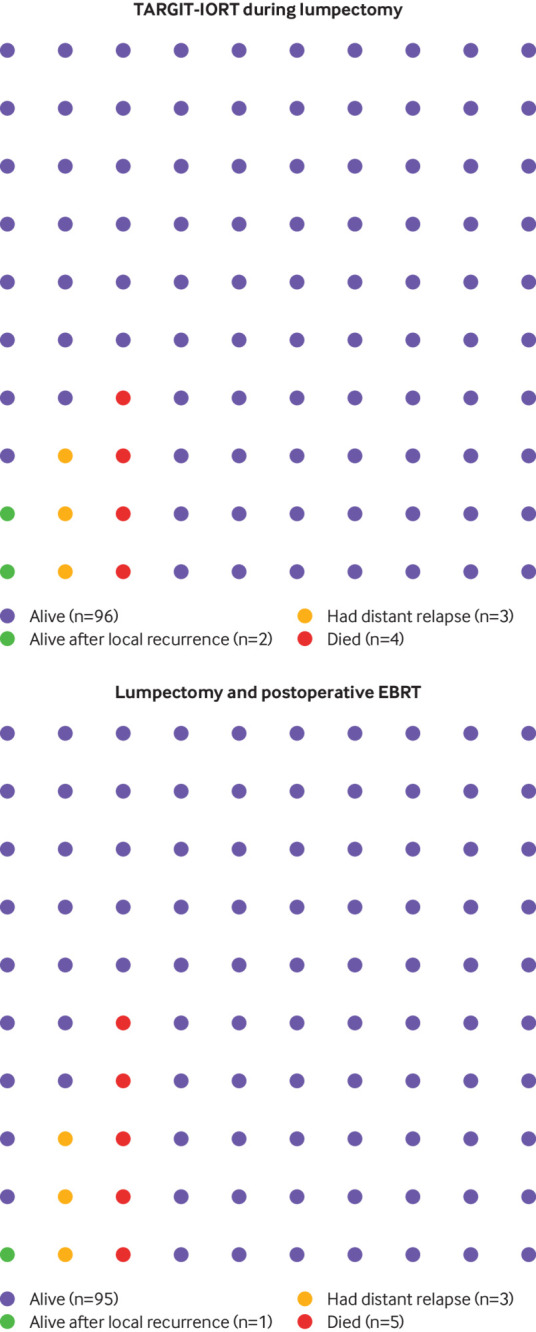
Pictogram showing outcomes in TARGIT-A trial of TARGIT-IORT *v* EBRT for breast cancer. Complete follow-up is available for five years. Each dot represents a patient. Absolute numbers of patients who had local recurrences, distant disease, and died (TARGIT-IORT: 24/1140 local recurrences, 34/1140 distant disease, and 42/1140 deaths; EBRT: 11/1158 local recurrences, 31 distant disease, and 56/1158 deaths) are apportioned per 100 patients for each treatment type. At five years, one more local recurrence and one less death were reported per 100 patients. EBRT=external beam radiotherapy; TARGIT-IORT=targeted intraoperative radiotherapy

The data presented here confirm that TARGIT-IORT is non-inferior to EBRT in terms of local control at protocol specified five year complete follow-up (local recurrence risk 2.11% for TARGIT-IORT *v* 0.95% for EBRT). Additionally, fewer deaths occurred with TARGIT-IORT. When we compared 1140 patients treated with TARGIT-IORT with 1158 patients treated with EBRT, 13 more local recurrences and 14 fewer deaths were reported. Figure 5 shows these raw data apportioned to 100 patients.

The Kaplan-Meier curves illustrate the long term results up to 12 years. These data confirm the comparable effectiveness of TARGIT-IORT versus EBRT in terms of cancer control, with no difference in local recurrence-free survival, invasive local recurrence-free survival, mastectomy-free survival, or distant disease-free survival for at least 12 years from randomisation (fig 3).

Breast cancer specific mortality was similar for both arms, however far fewer deaths were reported from causes other than breast cancer in the TARGIT-IORT arm. Even modern radiotherapy increases cardiac and lung cancer mortality and the results are consistent with our previously published data^48^ ^49^ ^50^ ^51^ ^52^ and a meta-analysis of randomised trials.^53^ ^54^ Furthermore, the Kaplan-Meier curve for overall survival for TARGIT-IORT always remains above EBRT, with the curves continuing to separate further well beyond 10 years.

### Strengths and weaknesses of the study

The pragmatic trial design is a major strength because the experimental arm simulated the potential future real world practice. Patients would get TARGIT-IORT during their initial cancer operation, and if found to have high risk factors, they would receive supplemental whole breast radiotherapy, making the results more clinically applicable. The international setting and broad inclusion criteria mean that the results are generalisable across relatively broad eligibility criteria and across various continents, even though centres of excellence participated in the trial.

**Fig 6:**
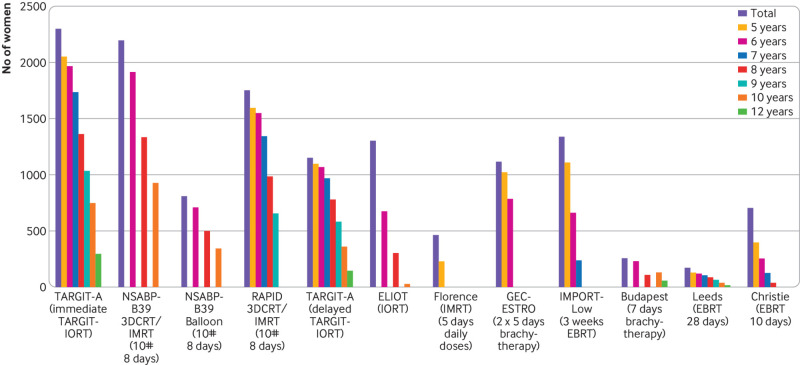
Amount of data in randomised trials of different techniques of partial breast irradiation for invasive breast cancer. 10# 8 days=10 fractions in eight days; EBRT=external beam radiotherapy; IMRT=intensity modulated radiotherapy; IORT=intraoperative radiotherapy; TARGIT-IORT=targeted intraoperative radiotherapy

Patients were randomised between 2000 and 2012. Substantial effort along with close collaboration from each centre enabled a high level of completeness of long term data. Consequently, another strength of the TARGIT-A trial is that it has more long term follow-up data than other published trials comparing individual techniques of partial breast irradiation with whole breast irradiation for invasive breast cancer (table 5, fig 6). Additionally, figure 2 shows that actual follow-up time is close to the follow-up time expected from the date patients were recruited, which means that substantial unknown data are unlikely. The long duration and high level of completeness of follow-up mean that the trial outcome is reliable and robust, and with 2298 participants, this trial is one of the largest in the field (table 5, fig 6). The trial was a result of an academic insight and was investigator initiated and funded by the HTA programme of the UK Department of Health and Social Care, rather than by industry sponsorship. The investigative team was multidisciplinary and consisted of patients and experts in surgical oncology, radiation oncology, clinical oncology, radiation physics, medical statistics, psycho-oncology, health economics, and clinical trial management.

**Table 5:** Number of patients at risk at various time points in published randomised trials that use different techniques of partial breast irradiation for invasive breast cancer

Study	Total	No of patients at risk*							
		5 years	6 years	7 years	8 years	9 years	10 years	11 years	12 years
TARGIT-A (immediate TARGIT-IORT)	2298	2048	1967	1736	1361	1035	749	587	295
TARGIT-A (delayed TARGIT-IORT)	1153	1097	1068	967	781	582	364	227	146
ELIOT (IORT)^15^	1305	—	676	—	305	—	29	—	—
Florence (IMRT; 5 days daily doses)^17^	520	260		—	—	—	—	—	—
GEC-ESTRO (2×5 days brachytherapy)^18^	1184	1081	829	—	—	—	—	—	—
IMPORT-Low (3 weeks EBRT)^19^	1343	1109	661	239	—	—	—	—	—
Budapest (7 days brachytherapy)^16^	258	—	231	—	113	—	134	—	57
NSABP-B39 3DCRT/IMRT (10# 8 days†)^20^	2193	—	1915	—	1335	—	929	—	—
NSABP-B39 Balloon (10# 8 days†)^20^	811	—	708	—	494	—	344	—	—
RAPID 3DCRT/IMRT (10# 8 days†)^21^	1754	1593	1548	1344	986	654	—	—	—
Leeds (EBRT 28 days)^14^	174	130	120	106	88	64	40	27	16
Christie (EBRT 10 days)^10^	708	400	250	127	40	—	—	—	—

10# 8 days=10 fractions in eight days; EBRT=external beam radiotherapy; IMRT=intensity modulated radiotherapy; IORT=intraoperative radiotherapy; TARGIT-IORT=targeted intraoperative radiotherapy.

Values are shown graphically in figure 6. Proportion of invasive cancer: 100% for TARGIT-A, ELIOT, IMPORT-Low, Budapest, Leeds, and Christie, 73% for NSABP-B39, 82% for RAPID, 89% for Florence, and 95% for GEC-ESTRO.

* Follow-up durations shown in Kaplan-Meier plots.

The ratio of ductal carcinoma in situ to invasive recurrence was higher in the TARGIT-IORT arm (12:32) compared with the EBRT arm (1:19; table 3). One limitation of this study is that we do not know if this finding is owing to overdiagnosis and ascertainment bias because of potentially more frequent use of mammography in patients randomised to TARGIT-IORT, or if it is a real effect. However, this increase in diagnosis of ductal carcinoma in situ in the TARGIT-IORT arm did not lead to a reduction in mastectomy-free survival.

Another limitation of the study was that we did not collect all the background risk factors for deaths from non-breast cancer causes. However, the major risk factors for cardiovascular disease^55^ and malignant disease^56^ that were formally collected during the trial were age and body mass index, and these factors were distributed evenly between the two randomised arms. While smoking history and other common risk factors were not collected, it is unlikely that their incidence would be imbalanced in such a large randomised trial. Additionally, cause of death could not be determined for all patients. Therefore, patients were deemed to have died of causes other than breast cancer only if the local principal investigator had clearly specified that the cause of death was not breast cancer and breast cancer was not present, and only when there was no record of the patient having had any relapse of breast cancer.

### The perspective in relation to other studies investigating partial breast irradiation

Partial breast irradiation was heralded as a new standard^12^ at the time of the first publication of the TARGIT-A trial.^11^ Several other supporting trials have since been published, including the ELIOT trial,^15^ and studies examining brachytherapy,^18^ and partial breast EBRT.^17^ ^19^ The TARGIT-A and ELIOT trials differ considerably in their inclusion criteria, and most importantly, have entirely different surgical and radiotherapeutic techniques, and so are not comparable. A Cochrane meta-analysis published in 2016^57^ included all diverse methods of partial breast irradiation, but could not make definitive conclusions because of data limitations. Our own meta-analysis that only examined mortality (initially published in 2016 and updated in 2018)^53^ ^54^ found that partial breast irradiation has no impact on breast cancer mortality but reduces non-breast cancer mortality and overall mortality.

In general, the other trials of partial breast irradiation with EBRT have included patients with cancer with considerably better prognosis. For example, when we compared the IMPORT-Low trial^19^ patient population with that of the TARGIT-A trial, 3% versus 22% had node positivity, and 9% versus 20% had grade 3 tumours. Furthermore, this new analysis of long term data suggests that the greater proportion of patients with higher risk disease has not jeopardised the outcome in the TARGIT-A trial. We recommend that risk adapted TARGIT-IORT should be used in patients who would have been eligible for the TARGIT-A trial. Partial breast irradiation with EBRT still requires up to three weeks of daily radiation with about 16 hospital visits.^19^ Although newer brachytherapy or some intensity modulated radiotherapy regimens could be completed in 10 fractions over five days, these trials had much smaller numbers of patients (1081^58^ and 260^17^ patients with five years of follow-up compared with 2048 in immediate TARGIT-A *v* EBRT; table 5, fig 6). Most of these techniques have adverse physical, social, financial,^59^ ^60^ ^61^ and environmental impacts,^43^ and do not substantially reduce the heavy workload of radiotherapy departments. Conversely, TARGIT-IORT delivered during the operation enables four fifths of patients to avoid visiting the radiotherapy centre at all.

### Meaning of the study and implications for clinicians and policy makers

The long term results of this trial have shown that risk adapted single dose TARGIT-IORT given during lumpectomy can effectively replace the mandatory use of several weeks of daily postoperative whole breast radiotherapy in patients with breast cancer undergoing breast conservation. Crucially, 80% of patients required no additional radiotherapy after TARGIT-IORT. Additionally, TARGIT-IORT reduced non-breast cancer mortality. The advantage to the patient of avoiding postoperative radiotherapy could be considered obvious. Furthermore, formal studies have also been performed and have reported quality of life and patient reported outcomes such as cosmesis, breast related quality of life, and breast pain to be superior with TARGIT-IORT in the first five years.^44^ ^45^ ^46^ ^47^ Additionally, patients prefer this approach even when faced with a potentially higher local recurrence risk.^28^ ^29^ ^30^ Moreover, 80% of patients, many of whom live a considerable distance from the radiotherapy centre,^43^ ^57^ avoid the need for daily hospital visits for three to six weeks, which would be required for established radiotherapy techniques. For such patients, TARGIT-IORT provides the opportunity for breast conservation rather than being obliged to choose mastectomy.^62^ Even as recently as 2015, in a modern urban community (New Jersey, US), patients who lived more than 9.2 miles from the radiation facility (or more than 19 minutes away by car) compared with less than 9.2 miles away were 36-44% more likely to receive a mastectomy than breast conservation.^63^

Another important advantage is the major cost savings for the health services reported in previously published studies of health economics of the TARGIT-A trial.^59^ ^60^ ^61^ All these factors are important when considering a change of policy and determining which treatments should be funded at the national level by organisations such as the NHS in the UK and Medicare or Medicaid in the US. While the payers will save scarce healthcare resources by using TARGIT-IORT, the providers will also want to use this approach when the payment model is changed to be value based rather than activity based.

Another important aspect is the well recognised phenomenon of overdiagnosis of breast cancer because of systematic population screening. This is a difficult problem because the potential of reduced breast cancer mortality needs to be balanced against the definite harms of overtreatment of women who might not have had a diagnosis of breast cancer if it were not for the screening programme. TARGIT-IORT could largely reduce the burden of treatment on such patients, and has been recommended by the Marmot committee.^64^

### Implications for patients

When these results are expressed from the patient’s perspective, without any definitions of non-inferiority, they would read as follows: “I understand from your explanation that if I choose to have intraoperative radiotherapy during my lumpectomy operation, the whole treatment will probably be completed in one go. I understand that the chance of avoiding a full course of traditional whole breast radiotherapy is about 80%, which requires several daily visits to complete. The results of this study have reassured me that choosing intraoperative radiotherapy doesn’t reduce my long term chances of survival or keeping my breast, and remaining cancer free. You have also told me that there will be fewer long term side effects, a better quality of life and that the cosmetic result is likely to be better. I am also reassured to learn that this treatment does in fact reduce my chance of death from causes other than breast cancer.”

Patients are entitled to choose which approach is right for them, based on effectiveness, convenience, personal cost, quality of life, and side effects. To allow a truly informed patient to make the choice between a risk adapted TARGIT-IORT policy and conventional EBRT, we need to supply the data using absolute numbers in an easily accessible and comprehensible way.^65^ A pictographic display (fig 4), based on the raw numerical data, is a transparent and accurate way of supporting the patient to make an informed choice.

We believe that the long term data presented in this paper, together with many benefits for the patient, provide compelling evidence in favour of TARGIT-IORT as an effective alternative for this large group of patients with early breast cancer who are suitable for breast conservation. Ultimately the treatment patients receive should be their choice and they should be provided with the data in a format which is transparent, straightforward, and easily understood.

### Future and ongoing work

Additional work based on these results includes subgroup analysis, an analysis of local recurrence as a hazard for distant disease, and an analysis exploring the mechanisms behind the differences in non-breast cancer mortality seen in the trial. We will also present a web based tool to allow clinicians to use the risk adapted approach. The inputs for this tool include individual patient data, and the output gives the probability of a patient needing supplemental EBRT after TARGIT-IORT within the TARGIT-A trial. Further investigation into the nature of local recurrences will include molecular markers and the location within the breast.

In the extended follow-up of the TARGIT-A trial (TARGIT-Ex; funded by the HTA programme of the National Institute for Health Research, Department of Health and Social Care in the UK, HTA 14/49/13) we will use new methods such as direct patient contact and linkage with the Office for National Statistics. We are also currently inviting women who would fall outside the eligibility criteria of the TARGIT-A trial to participate in the TARGIT-B(oost) trial (funded by HTA 10/104/07), already opened in 36 centres internationally, which is comparing TARGIT-IORT as a tumour bed boost with EBRT boost in younger women or women who have a higher risk of disease to test for superiority in terms of local control and survival.

**What is already known on this topic**

When early breast cancer is treated with breast conserving surgery (lumpectomy) rather than mastectomy, adjuvant whole breast postoperative external beam radiotherapy, given as multiple doses over several days, reduces the risk of local recurrence
Restricting radiotherapy to only the area around the tumour by using intraoperative radiotherapy has the benefits of precision and immediacy, and avoids the inevitable delay in starting postoperative radiotherapy
Early results of using single dose targeted intraoperative radiotherapy (TARGIT-IORT) during lumpectomy indicate this approach has many advantages for the patient, such as less travelling for treatment, improved quality of life, and fewer side effects

**What this study adds**

The results of the TARGIT-A trial show that TARGIT-IORT has similar long term local control and cancer survival outcomes to whole breast radiotherapy
Mortality from other causes was lower in the TARGIT-IORT arm
Single dose TARGIT-IORT during lumpectomy should be accessible to healthcare providers and discussed with patients when surgery for breast cancer is being planned

## Web extra

Extra material supplied by authors

Web appendix: e-Figures

## Notes

Contributors: JSV, MBa, and JST were responsible for trial concept, trial design, trial management, data interpretation, and writing of the manuscript. FW, DJJ, JST, MBa, and JSV contributed to trial concept, trial design, trial management, training and accreditation of centres, patient accrual and treatment, data collection, data interpretation, and writing of the manuscript. JSV, FW, JST, MBa, SM, HLF, MD, ES, MS, and MA contributed to training and accreditation of centres, patient accrual and treatment, data collection, and writing of the manuscript. JSV, MBa, MBu, and NRW designed the statistical analysis plan and contributed to statistical analysis, trial coordination, data collection, data interpretation, and writing of the manuscript. CS and TC contributed to trial design, patient accrual, patient treatment, data interpretation, and writing of the manuscript. WE, MD, LV, FB, MP, GG, WP, KJD, MN, JB, GP, and MS contributed to patient accrual, patient treatment, data collection, and data interpretation. SM, WE, SP, JSV, ES, MS, MLB, DH, SLa, SLu, DM, RH, PK, and DB contributed to setting up their centres, patient accrual, treatment, and data collection. JSV was involved with design and preclinical tests of the Intrabeam system. CB-G, NR, and IP contributed to training, trial coordination, trial management, data collection, and writing of the manuscript. MF advised about pathological aspects of the trial. MBe reviewed the results and their interpretation, provided patient perspective and contributed to the manuscript. The authors take full responsibility for the manuscript. The initial draft was written by JSV, MBu, JST, and MBa, who revised it by following comments from all other authors and are the guarantors. The chief investigator/corresponding author and the trial statistician had access to all data sent by the trial centre for analysis; all authors were responsible for decision to submit the manuscript. The corresponding author attests that all listed authors meet authorship criteria and that no others meeting the criteria have been omitted.

Ethical approval: The study received ethics approval from the joint University College London and University College London Hospital committees of ethics of human research (99/0307).

Data sharing: University College London is supportive of data sharing and will endeavour to assist in requests for data sharing. All requests for data sharing will adhere to the UCL Surgical and Interventional Trials Unit (SITU) data sharing agreement policy. These data will be held at UCL on secure servers and cannot be released to any third parties. All requests for access to the data and statistical code will be formally requested through the use of a SITU data request form which will state the purpose, analysis and publication plans together with the named collaborators. All requests are dealt with on a case-by-case basis. All requests will be logged and those successful will have a data transfer agreement which will specify appropriate security and privacy agreements, and acknowledgment of the TARGIT Trialists’ Group, investigators, the sponsor, and funders.

The lead author affirms that the manuscript is an honest, accurate, and transparent account of the study being reported; that no important aspects of the study have been omitted; and that any discrepancies from the study as planned (and, if relevant, registered) have been explained.

Dissemination to participants and related patient and public communities: We plan to widely disseminate the published paper. We shall use all modern media and engage patients and our own institutional public relations departments.
