# Supplementary material for: Long term survival and local control outcomes from single dose targeted intraoperative radiotherapy during lumpectomy (TARGIT-IORT) for early breast cancer: TARGIT-A randomised clinical trial
Source: BMJ. 2020 Aug 19;370:m2836. doi: 10.1136/bmj.m2836 (PMC7500441; doi:10.1136/bmj.m2836)

e-Figure 1 The TARGIT technique of targeted intraoperative radiotherapy for breast cancer. The Intrabeam device (left) and a schematic diagram showing how the spherical applicator is inserted and positioned into the tumour bed (right). For further details see targit.org.uk

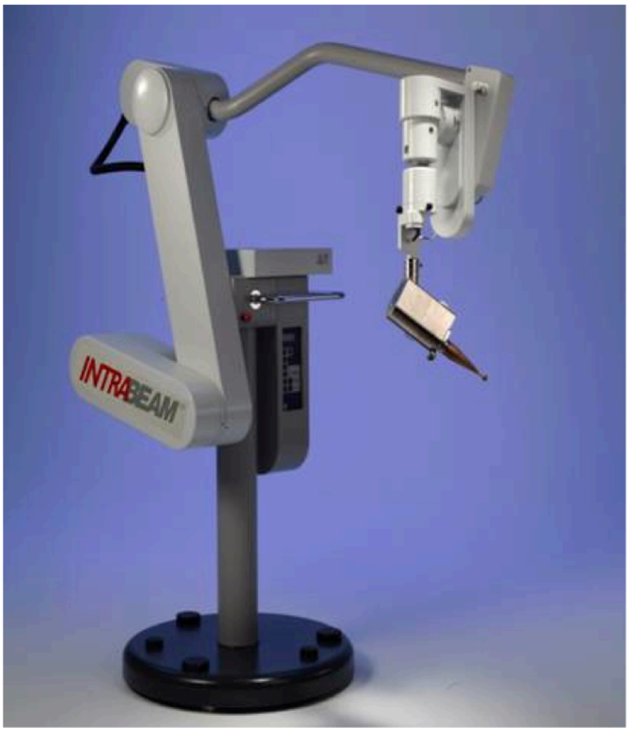

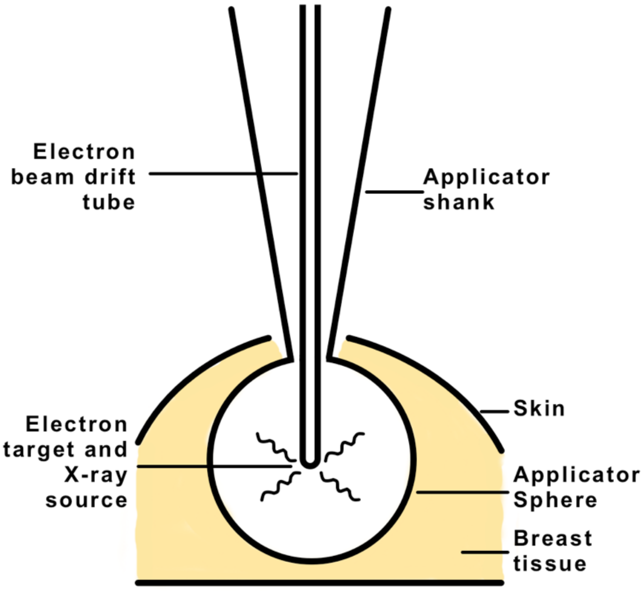

eFigure 2 Forest plot of hazard ratios of local recurrence-free survival as per country, which shows that there is no heterogeneity between countries.

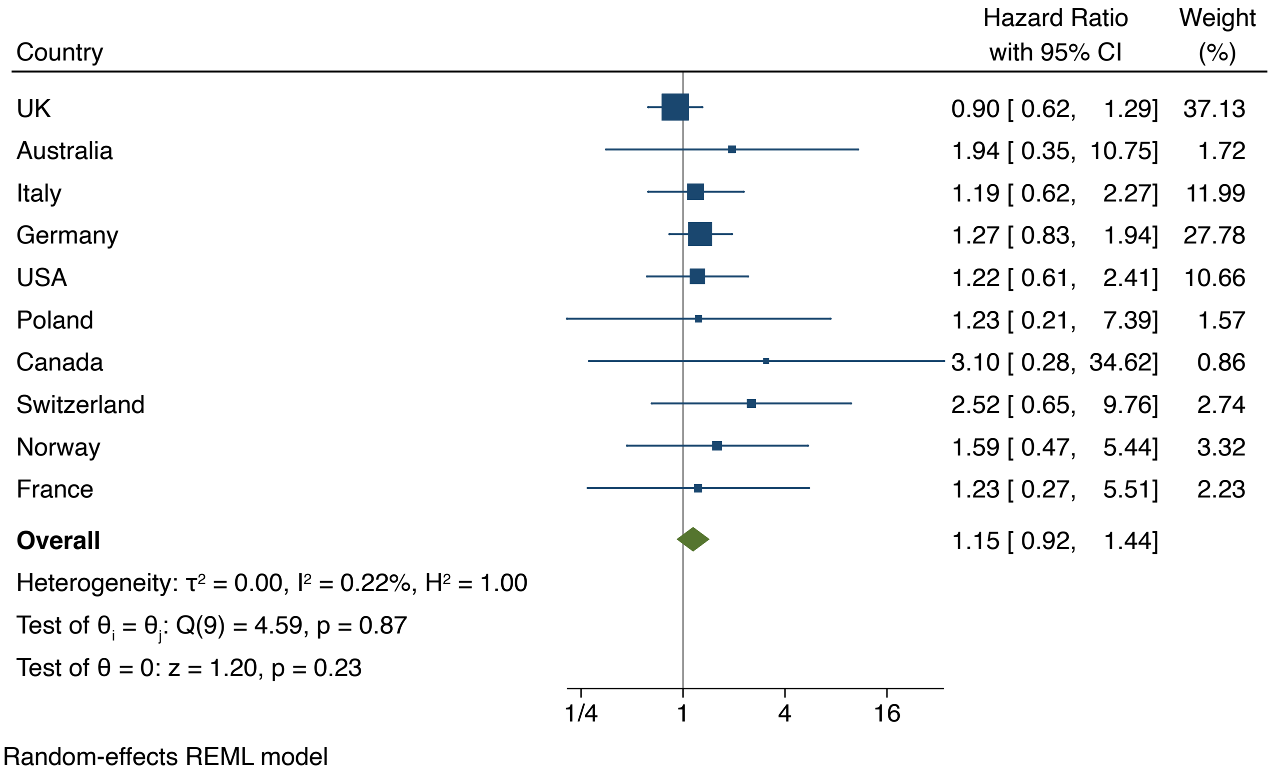

Supplement: Supplementary file 1 — Web appendix: e-Figures [file vaij055562.ww.pdf]
